# Effects of season, catching method, and thinning on carcass quality and production parameters in 4 different broiler production systems in the Netherlands

**DOI:** 10.1016/j.psj.2024.103688

**Published:** 2024-03-22

**Authors:** R.A. van Emous, J. van Harn, J.W. van Riel

**Affiliations:** Wageningen Livestock Research, De Elst 1, Wageningen, WD NL-6708, the Netherlands

**Keywords:** broiler, slow-growing, production systems, carcass quality

## Abstract

In the Netherlands, poultry meat production has been evolving in recent years toward broiler production systems with slow-growing broilers. In this study the effects of season, catching method, and thinning on carcass quality and production parameters in four different broiler production systems in the Netherlands was evaluated. The data for this study were collected from slaughterhouse data in 2018, 2019, and 2020 and contained information about four different broiler production systems: conventional (fast growing) broilers (**CONV**), 2 different indoor slow-growing broilers (**SGB1** and **SGB2**), and Better Life 1 Star (**BLS**) concept with slow-growing broilers. The data set consisted of 14,976, 1,730, 3,713, and 1,121 records (flocks) for CONV, SGB1, SGB2, and BLS, respectively. All three production systems with slow-growing broilers had a lower slaughter weight, average daily gain (**ADG**), first-week mortality, and total mortality than CONV (no-thinning). ADG of SGB2 and BLS was lower than that of SGB1. Slaughter weight and ADG were the lowest when day-old chicks were placed in March/April and the highest when they were placed in September/October. All slow-growing broiler production systems had a lower footpad lesion score and a lower incidence of hock burns, leg hematomas, breast hematomas, dead-on-arrival chickens (**DOA**), and total rejects than CONV. Autumn flocks had more hock burns, a higher footpad lesion score, and more wing hematomas than spring flocks. More scabby hips and fewer total rejects were found during the summer months than during the winter months. Thinning flocks had more scabby hips, ammonia burns, and DOAs and fewer hock burns, footpad lesions, wing hematomas, leg hematomas, breast hematomas, and total rejects than no-thinning flocks. Mechanically caught flocks had more ammonia burns, DOAs, and total rejects than manually caught flocks. In conclusion, this study showed that all three production systems with slow-growing broilers had a lower first-week mortality and total mortality and better scores on most carcass quality parameters and welfare indicators than CONV.

## INTRODUCTION

Most of the broilers in Europe are fast-growing broilers kept indoors at stocking densities of 33 to 42 kg/m^2^. This conventional broiler production system is, however, under discussion because of animal welfare issues. Initiated by societal debates, especially in Northwestern Europe, poultry meat production is in transition toward production systems with slow-growing broilers with the aim to improve broiler welfare (e.g., Bergmann et al., 2016; Saatkamp et al., 2019; de Jong et al., 2022). In 2007, the Better Life 1 star label (“Beter Leven keurmerk”) for broilers was introduced in the Netherlands. In this system, broilers are of slow-growing breeds (max. 45 g/d), housed at a maximum stocking density of 25 kg/m^2^, with 3% natural daylight, enrichments (bales, grains distributed in the litter), and have access to a covered range (veranda), which is specific for this broiler production system. Since 2014, Dutch supermarkets have switched to offering fresh poultry meat exclusively from slower-growing broilers, either from Better Life 1 Star label (**BLS**) or other concepts with slower-growing broilers. In other concepts, broilers of slow-growing breeds (45–50 g/d) are housed at stocking densities between 30 and 38 kg/m^2^ in houses with, in some cases, daylight, and environmental enrichment, such as straw/lucerne bales, and/or scattered grain (Saatkamp et al., 2019). In 2021, all the Dutch retailers committed to only selling fresh poultry meat from BLS broilers from 2023 at the latest (Dierenbescherming, 2021). Due to the transition in the supermarkets to these types of broiler production systems, the production of slow-growing broilers in the Netherlands increased between 2014 and 2017 from just 25,000 to about 2,7 million broilers per week. At the end of 2017, approximately 90% of all fresh poultry meat in Dutch supermarkets originated from slow-growing broilers. In 2021, this led to approximately 40 to 45% of all broilers in the Netherlands were kept according to an alternative production system, characterized by the use of slower-growing breeds (van Horne and Vissers, 2022). In Europe, more countries (Belgium, Denmark, and Germany) discussed the implementation of slow-growing production systems (Bergmann et al., 2016; Politiken, 2020).

There are indications from practice that slow-growing broilers can show more skin damage (such as skin scratches and bruises), which could have potentially negative effects on welfare. This may be due to the fact that slow-growing broilers are more active throughout their life cycle (Bokkers and Koene, 2003; Wilhelmsson, 2016; Rayner et al., 2020) and are kept under different conditions (lower stocking density, longer uninterrupted dark period, higher light intensity, etc.). The occurrence of skin scratches is related to the distance between chickens, for example, during a fear reaction or due to feed competition (Fallavena, 2005). Injuries such as broken bones, bruises, or dislocations usually occur mainly during the catching, loading, and transport of the broilers (Chauvin et al., 2011; Jacobs et al., 2017; Kittelsen et al., 2017). It is assumed that slow-growing broilers, especially when they are caught during daylight, are more active during catching, which can cause more injuries (Gerritzen et al., 2019). The latter authors found more wing hematomas and wing dislocations in slow-growing than in conventional fast-growing broilers.

Despite the rapid increase in the market share of slow-growing broilers, there is still a lack of specific knowledge about slow-growing broilers on many topics within the production chain. Most all scientific and applied knowledge is based on research with conventional (fast-growing) broilers, although there is increasing interest in studying slower-growing broilers. In addition to the lack of specific knowledge about resource efficiency, behavior, environment, and health, very little is known about the carcass quality of slow-growing broilers. Therefore, a study was conducted to evaluate the effects of season, catching method, and thinning on carcass quality and production parameters in four different broiler production systems in the Netherlands. For this study, data were collected from a Dutch slaughterhouse with two locations. More insights into these parameters can support the further development of slow-growing production systems to an even more welfare-friendly and efficient production chain.

## MATERIALS AND METHODS

### Broiler Production Systems

The data set consists of the following four broiler production systems:

1. Conventional (**CONV**): indoor broiler production system with a concrete floor (with bedding material), using conventional fast-growing broilers with an ADG of at least 60 g/d, a slaughter age between 32 (thinning) and 46 d (no-thinning), and a maximum stocking density of 42 kg/m^2^ according to the EU Broiler Directive (2007/43/EC), without environmental enrichment and natural light.
2. Slow-growing broilers 1 (**SGB1**): indoor broiler production system with a concrete floor with bedding material and natural light entrance (3% of the floor space), using a slow-growing broiler strain with a maximum ADG of 50 g/d, a maximum stocking density of 34 kg/m^2^, 1 bale (straw, hay, lucerne, or other manipulable material) per 1,000 broilers, scattered feed, plant based diet, Round Table on Responsible Soy (RTRS) soy, 70% grain, and a minimum slaughter age of 47 d.
3. Slow-growing broilers 2 (**SGB2**): indoor broiler production system (concrete floor with bedding material) with natural light entrance (3% of the floor space), using a slow-growing broiler strain with a maximum ADG of 45 g/d, a maximum stocking density of 30 kg/m^2^, 1 bale (straw, hay, lucerne, or other manipulable material) per 1,000 broilers, scattered feed, plant based diet, RTRS soy, 70% grain, and a minimum slaughter 49 d.
4. Better Life 1 Star (**BLS**): broiler production system based on the extensive indoor system as described by the EU with an indoor house (concrete floor with bedding material) with natural light entrance (at least 3% of the floor space) and, in addition, a covered range (veranda), using a slow-growing broiler strain with a maximum ADG of 45 g/d, a minimum slaughter age of 56 d, a maximum stocking density of 25 kg/m^2^, 2 g of scattered feed/grain per chicken per day, and 1 bale (straw, hay, lucerne, or other manipulable material) per 1,000 broilers. These standards are according to the criteria of the Better Life 1 Star label of the Dutch Society for the Protection of Animals (Dierenbescherming, SPA).

### Data Sources

The data for this study were collected in 2018, 2019, and 2020 and originated from a Dutch slaughterhouse with two locations. The data set contained carcass quality data from four different broiler production systems: CONV, SGB1, SGB2, and BLS. The data set comprised the following production parameters: slaughter age (days), slaughter weight (g), first-week mortality (%), and total mortality (%). ADG was calculated by dividing slaughter weight by slaughter age. The data set also included information about slaughter date, the hatchery, feed company, catching crew, and catching method (manual or mechanical). For CONV broilers, a distinction was made between thinning flocks (part of the broiler flock delivered to the slaughterhouse before the end of the growing cycle) and no-thinning flocks (broilers that are delivered at the end of the growing cycle). Thinning is not allowed in the abovementioned production systems with slow-growing broilers. For the carcass quality data, the standardized assessment system for broilers at the slaughter line in the slaughterhouse was used, which is an important part of the Dutch IKB Chicken certification scheme (IKB Kip, 2017). Carcass quality parameters were standardly assessed by government veterinarians at the slaughterhouse and expressed as a percentage of the total number of assessed birds. At the slaughterhouse, at least 100 broilers from each poultry transport vehicle (length approx. 15 m) with a container system with approx. 22 containers and in total approx. 7,000 broilers (Meyn Evo, Zaandam, the Netherlands) were randomly assessed, 50 broilers at approximately 1/3 of the semi-trailer and another 50 broilers at approximately 2/3 of the semi-trailer. The following parameters were assessed and described below: hock dermatitis/burns, footpad lesions, scabby hips, wing hematomas, leg hematomas, breast bleedings, ammonia burns, dead-on-arrival chickens (**DOA**), and total rejects. All the previous mentioned parameters can also be seen as welfare indicators.

### Hock Dermatitis or Hock Burns

Hock burns are identified as black-brown discolorations around the hock joint. Each chick was scored on both legs before the legs were cut off, with score 0 = *no deviation* and score 1 = *deviation* (black-brown discoloration greater than 0.5 cm^2^).

### Footpad Lesions

Footpad lesions are a condition that causes necrotic lesions on the plantar surface of the footpads of growing broilers. The slaughterhouse assesses 100 (left or right) legs per flock and gives the following score per leg: 0 = *no lesion*, 1 = *mild lesion*, and 2 = *severe lesion*. This score was calculated as follows: 100% × ((0.5 × the total number of birds with score 1) + (2 × the total number of birds with score 2))/the total number of scored birds. The footpad score (**FPS**) can range from 0 (all birds having no lesions) to 200 (all birds having score 2).

### Scabby Hips

Scabby hips involve damage and/or flaking of the skin on the back and/or thigh. The damage occurs in the form of scratches and/or injuries or scabs in various stages of recovery. The scratches are caused by chickens crawling or walking over each other and scratching the skin with their nails. The assessment threshold for scratches was three scratches longer than 2 cm, and the threshold for injuries/scabs was a clearly visible opening of the skin.

### Wing Hematomas

Wing hematomas manifest themselves as a discoloration in or under the skin due to bleeding from blood vessels, with or without bone fractures. This is with the exception of the last part of the wing and the wing tip. Wing hematomas are caused by bruises, fractures, or dislocations in the living animal and can occur during the catching and transport of the broilers or during the tilting of the containers at the slaughter plant. The threshold for wing hematomas was formulated as a bruise or lesion larger than 2 cm^2^.

### Leg Hematomas

Leg hematomas involve a discoloration in or under the skin on the drum and/or thigh. This is caused by bleeding from blood vessels, with or without bone fractures. This is with the exception of bruises above the hock joint, which are a result of hanging up on the slaughter hooks. The threshold for leg hematomas was formulated as a discoloration greater than 1 cm^2^.

### Breast Bleedings

Breast bleeding is a discoloration in or under the skin and/or in the breast meat due to bleeding from blood vessels. The threshold for breast bleedings was formulated as a discoloration greater than 1 cm^2^.

### Ammonia Burns

Ammonia burns are local swellings (mostly on the breast) with damage to the skin (crust/crater), with or without discoloration. These spots are caused by prolonged skin contact with poor-quality litter. The threshold for ammonia burns was more than one discolored ammonia burn or more than three pale ammonia burns.

### Dead-On-Arrival

Dead-on-arrival chickens are all dead chickens that are delivered dead to the slaughterhouse.

### Total Rejects

Total rejects include the following chick abnormalities: abnormal color/odor/taste, polyserositis, arthritis/synovitis, hepatitis, *ornithobacterium rhinotracheale* (**ORT**), back muscle inflammation, pericarditis, ascites syndrome, subcutaneous inflammation, wooden breast, rejected parts, and other abnormalities.

### Number of Records and Data Set

The original data set consisted of a total of 22,858 records divided over the years (2018, 2019, and 2020) and broiler production systems. However, due to missing data, the number of records for the final analysis of the various parameters was 21,540. A total of 14,976 records were available for the CONV broilers, of which 7,585 records were available for thinning flocks and 7,391 records for no-thinning flocks. A total of 1,730, 3,713, and 1,121 records were available for the analysis of SGB1, SGB2, and BLS, respectively.

Various broiler breeds have been used over the years within the different broiler production systems. For the CONV broilers, mainly Ross 308 broilers (as-hatched) were used (95%), the remaining 5% consisted of Cobb 500 (as-hatched) or by-products (i.e. Cobb and Ross 308 males from the female lines and Cobb and Ross 308 females from the male lines). The majority (98.4%) of the SGB1 broilers (as-hatched) were Hubbard JA287, JA787 and JA987 broilers, while the remainder consisted of Ranger Classic (1.6%). For the SGB2 and BLS production systems, only Hubbard JA257 and JA757 broilers (as-hatched) were used.

### Statistical Analysis

All analyses were performed using Genstat software (version 19), where at *P* ≤ 0.05 a difference was considered statistically significant and at 0.05 < *P* ≤ 0.10 a difference was considered a trend. A mixed model (**REML**) analysis was performed to simultaneously estimate the fixed effect of season (by Fourier transformation according to Yassin et al., 2009); the effects of broiler production system, slaughter location, and mechanical catching; and the effect of the random coefficients of variance (i.e., the relative contribution of a list of possible effects) of the carcass quality traits. For the production system CONV, the fixed effect of thinning was also estimated. The random coefficients of variance include the total rest variance, which is the variance that could not be explained by the effects of season, production system, slaughter location, and catching method. This results in a quantification of the rest of the variance sources: farm effects; system-specific effects of feeding company, hatchery, breed, farm, and slaughter location; farm-specific effects of broiler house, starting date (of new chickens), season, slaughter location, and season and system; broiler house-specific effects of starting date and catching team; and catching team-specific season effects (see below). All traits, except footpad lesions, were log+0.1 transformed. Footpad lesions were square-root transformed.

The statistical model was as follows:

$$Y_{ijklmnopqvw}=\left(\beta_{0ij}+\beta_{0k}\right)+\left(\beta_{1k}+{\underset{‾}{\varepsilon }}_{\beta 1l}\right)*X_{1}+\left(\beta_{2k}+\varepsilon_{\beta 2v}\right)*X_{2}+\beta_{3}*X_{3}+{\underset{‾}{\varepsilon }}_{ik}+{\underset{‾}{\varepsilon }}_{im}+{\underset{‾}{\varepsilon }}_{in}+{\underset{‾}{\varepsilon }}_{io}+{\underset{‾}{\varepsilon }}_{il}+{\underset{‾}{\varepsilon }}_{l}+{{\underset{‾}{\varepsilon }}_{l}}_{p}+{{\underset{‾}{\varepsilon }}_{l}}_{q}+{{\underset{‾}{\varepsilon }}_{l}}_{k}+{\underset{‾}{\varepsilon }}_{lpq}+{\underset{‾}{\varepsilon }}_{v}+{\underset{‾}{\varepsilon }}_{kw}+{\underset{‾}{\varepsilon }}_{ijklmnopqvw}$$

with the following parameters and variables:

*β_0ij_, β_0k_, β_3_*: the fixed intercept of system *i* and thinning effect *j*, the fixed effect of location *k*, and the fixed effect of mechanical catching.

*β_1k_, β_2k_*: parameters for the effect of season and location *k* (after Fourier transformation of calendar day number; see below).

X_1_, X_2_: sinus $\left(\frac{2\pi }{365}*d\right)$ and cosinus $\left(\frac{2\pi }{365}*d\right)$, with *d* being the day number in a year (based on the date of birth of the broilers, thus from 1 to 365). The seasonal sinus wave represents the spring–autumn variation (March 1 vs. September 1); the seasonal cosinus wave represents the summer–winter variation (January 1 vs. June 1).

X_3_: dummy variable for mechanical catching.

*ε_β1l_, ε_β2v_*: random regression effects of farm-specific and catching team-specific seasonal effects.

*ε_ik_, ε_im_, ε_in_, ε_io_, ε_il_*: random coefficients of slaughter location *k*, feeding company *m*, hatchery *n*, breed *o*, and farm *l* (all within system *i*).

*ε*_l,_*ε*_lp,_*ε*_lq,_*ε*_lk,_*ε*_lpq_: random coefficients of broiler farm *l* and house *p*, starting date *q*, slaughter location *k,* and house-specific starting date *pq* (all within farm *l*)*.*

*ε*_v,_*ε*_kw_: random coefficients of catching team *v* and specific combination of slaughter location and week number *kw.*

## RESULTS AND DISCUSSION

### Production Performance

In the data set, CONV, SGB1, SGB2, and BLS broilers had an average slaughter age of 41.7, 49.5, 53.3, and 56.4 d, respectively (Table 1). This corresponds well with the age criteria set for the different broiler production systems with slow-growing broilers, namely 47, 49 to 55, and 56 d for SGB1, SGB2, and BLS, respectively.

**Table 1:** Average slaughter age (days), body weight (g), body weight gain (g/d), first-week mortality (%), and total mortality (%) of the different broiler production systems.^1^

	CONV	SGB1	SGB2	BLS	*P*-value
Slaughter age (days)	41.7	49.5	53.3	56.4	
Slaughter weight (g)	2,640^a^	2,368^b^	2,346^b^	2,398^b^	<0.001
Body weight gain (g/d)	63.3^a^	47.8^b^	44.0^c^	42.5^c^	<0.001
First-week mortality (%)	1.08^a^	0.73^b^	0.77^b^	0.71^b^	<0.001
Total mortality (%)	2.89^a^	2.12^b^	1.76^b^	1.70^b^	<0.001

a-c Means within a column with no common superscript differences (*P* ≤ 0.05).

1 CONV: conventional fast-growing broilers; SGB1: slow-growing broilers 1; SGB2: slow-growing broilers 2; BLS: better life 1 star broilers.

The slaughter weight of CONV broilers (2,640 g; no-thinning flocks) was considerably higher (*P* < 0.001) than the average slaughter weight of SGB1 (2,368 g), SGB2 (2,346 g), and BLS broilers (2,398 g). In the data set of CONV, 95.0% of all flocks were the Ross 308 breed, and 5.0% were so-called by-products, that is, male chickens from the Ross 308 and Cobb 500 female lines and female chickens from the Ross 308 and Cobb 500 male lines. For the three different slow-growing broiler production systems, mainly Hubbard JA257, JA757, JA787, JA287, and JA987 chickens were used, which have lower growth performance characteristics than the fast-growing breeds used for CONV. There was no difference in slaughter weight between the slow-growing broiler production systems.

The ADG of SGB1, SGB2, and BLS was 47.8, 44.0, and 42.5 g, respectively, which remained below the criteria of maximum ADG of 50.0, 45.0, and 45.0 g. ADG (63.3 g) was significantly higher (*P* < 0.001) for the CONV broilers than for the slow-growing broiler production systems, which was caused by the previously mentioned difference in breeds used. The SGB1 broilers had a significantly higher ADG than both the SGB2 and BLS broilers, which is caused by differences in breeds and production system criteria for ADG. For the SGB1 production system, mainly Hubbard JA287, JA787, and JA987 were used, which had higher growth characteristics than Hubbard JA257 and JA757, which were used for the SGB2 and BL1S production systems.

First-week and total mortality was significantly higher for the CONV broilers than for the three slow-growing broiler production systems (*P* < 0.001). This is in agreement with research by Castellini et al. (2002) and Dal Bosco et al. (2014), who found a higher mortality in fast-growing than in slow-growing broilers raised under organic conditions. Two decades ago, Van Horne et al. (2003) already found a lower mortality rate in slow-growing broilers than in fast-growing broilers (1.5% vs. 5.6%). The higher mortality in conventional broilers can partly be explained by the fact that conventional fast-growing broilers are somewhat less robust than slow-growing broilers (van Horne et al., 2003). This difference in robustness is also reflected in lower antibiotic use in slow-growing broilers (de Jong and van Riel, 2020). Slow-growing broilers also have a lower risk of failure due to heart and circulatory disorders (sudden death syndrome, heart failure syndrome, hydrops ascites) than fast-growing broilers (van Horne et al., 2003). In the present study, no differences were found in first-week and total mortality between the three slow-growing broiler production systems. The total mortality of CONV, SGB1, SGB2, and BLS broilers was, on average, approximately 25% lower than the Dutch standards for these different broiler production systems: 3.5, 3.0, 3, and 2.5%, respectively (KWIN, 2021).

For thinning and no-thinning flocks (CONV broilers), average slaughter age was respectively 34.5 and 41.7 d, average slaughter weight was 1,966 and 2,640 g, and average ADG was 57.0 and 63.3 g (Table 2). Although no-thinning flocks grow over a longer period of time, there was no significant difference in first-week and total mortality between thinning and no-thinning flocks. This may be caused by the fact that the thinning and no-thinning flocks were not always delivered to the same slaughter plant.

**Table 2:** Average slaughter age (days), body weight (g), body weight gain (g/d), first-week mortality (%), and total mortality (%) of CONV thinning flocks and no-thinning flocks.^1^

	CONV thinning flocks	CONV no-thinning flocks	*P*-value
Slaughter age (days)	34.5	41.7	
Slaughter weight (g)	1,966^b^	2,640^a^	<0.001
Body weight gain (g/d)	57.0^b^	63.3^a^	<0.001
First-week mortality (%)	1.07	1.08	0.97
Total mortality (%)	2.85	2.89	0.83

a-b Means within a column with no common superscript differences (*P* ≤ 0.05).

1 CONV: conventional fast-growing broilers.

### Seasonal Effects on Production Performance

**Figure 1:**
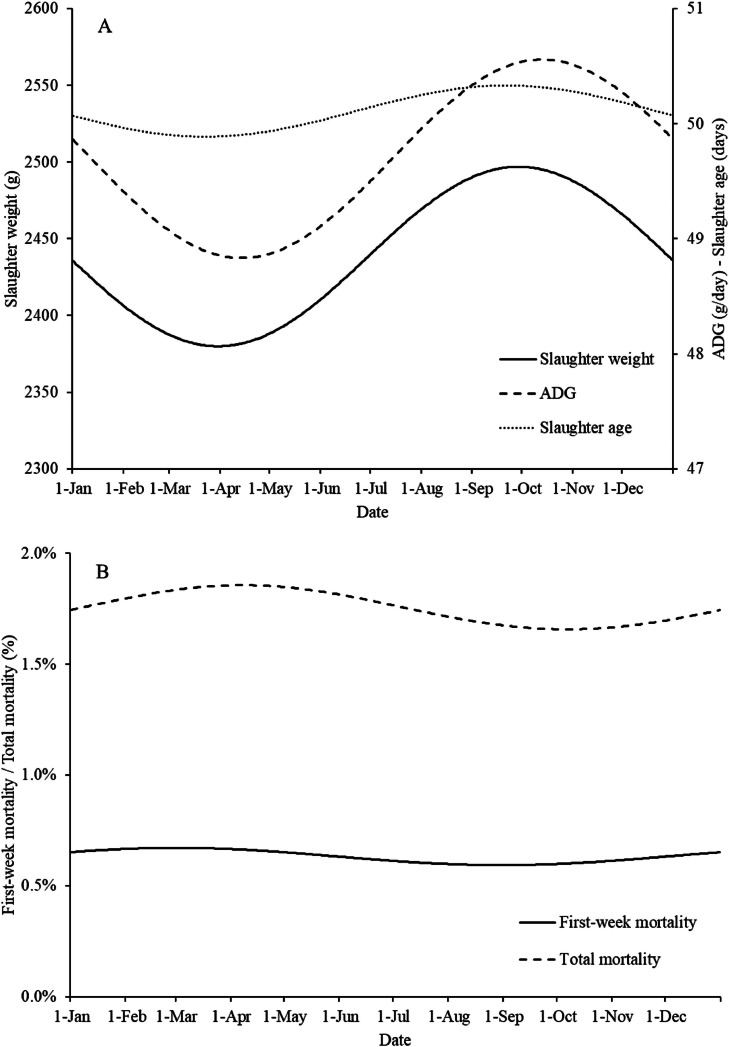
Estimated seasonal effects for slaughter weight, ADG, slaughter age (A), first-week mortality, and total mortality (B) of the broiler flocks. The X-axis represents the day of the year when DOCs were placed in the broiler house, starting on January 1 (d 1).

Significant effects of season were found on slaughter age, slaughter weight, ADG, first-week mortality, and total mortality (Table 3). Slaughter weight was the lowest when day-old chicks (**DOC**) were placed in late March/early April and the highest when DOCs were placed at the end of September/early October (Figure 1A). ADG was the lowest when DOCs were placed in the broiler house in April and the highest when DOCs were placed in October. The lower slaughter weight and BW gain of DOCs placed in April may be due to the large fluctuations that occurred between day and night temperatures. A rapid increase in temperature has a decreasing effect on feed intake and therefore indirectly on BW gain and slaughter weight (Leeson and Summers, 2005). When DOCs were placed in October, the fluctuations in day and night temperatures were smaller, which can lead to a more constant climate in the house.

**Table 3:** Estimates of the fixed effects on broiler flock level in the statistical model of season per production performance parameter.

	*β_1_* Season (sinus)^1^	*Β_2_* Season (cosinus)^2^
Slaughter age (days)	-3.10 * 10^−1^†	-3.42 * 10^−2^
Slaughter weight (g)	-68.77†	-0.86
Body weight gain (g/d)	-0.97†	0.18
First-week mortality (%)	4.49 * 10^−2^†	2.70 * 10^−2^†
Total mortality (%)	5.25 * 10^−2^†	-3.16 * 10^−4^

† Significant effect *(P* ≤ 0.05); where no superscript is provided, no significant effect or trend was found.

1 *β1:* The seasonal sinus wave represents the spring–autumn variation (March 1 vs. September 1).

2 *β2:* The seasonal cosinus wave represents the summer–winter variation (January 1 vs. June 1).

The seasonal effect on first-week mortality and total mortality was less pronounced than the seasonal effect on slaughter weight and ADG (Figure 1B). First-week mortality was the highest when DOCs were placed in late March/early April and the lowest when DOCs were placed in late August/early September. Total mortality was the highest when DOCs were placed between mid-March and late April and the lowest when DOCs were placed between mid-September and late October.

### Carcass Quality, and Welfare Indicators

The SGB1, SGB2, and BLS broilers had significant lower levels of hock burn, footpad lesion scores, leg hematomas, breast bleedings, DOAs, and total rejects than the CONV broilers (Table 4). No significant differences in ammonia burns, scabby hips, and wing hematomas were found between the different broiler production systems. A higher prevalence of hock burns and a higher footpad lesion score in conventional broilers were also observed by Wilhelmsson (2016) and Rayner et al. (2020). In the study by Wilhelmsson (2016), 58% and 26% of hock burns and 59% and 5% of footpad lesions were found in Ross 308 and Rowan Ranger broilers at 9 wk of age, respectively. They concluded that the wetter litter in houses with Ross 308 was the cause for the higher prevalence of hock burns and footpad lesions. Rayner et al. (2020) compared slow-growing broilers (ADG of 45–49 g/d) with conventional fast-growing (>60 g/d) broilers. At 6½ and 5 wk of age, 14.6% and 26.7% hock burns and 0.2% and 7.3% footpad lesions were observed in slower and fast-growing broilers, respectively. The higher percentage of hock burns and footpad lesions in conventional fast-growing broilers can be explained by the lower activity (Bokkers and Koene, 2003; Wilhelmsson, 2016; Rayner et al., 2020), higher feed intake (and thus higher excretion) (Wilhelmsson, 2016), and higher stocking density. This combination of factors can result in poorer litter quality, affecting the hocks and footpads (Wilhelmsson, 2016; Rayner et al., 2020). The higher percentage of leg hematomas in conventional broilers may be due to poorer muscle development, which in turn is due to the lower bird activity (Bokkers and Koene, 2003; Wilhelmsson, 2016; Rayner et al., 2020), increasing the risk of leg damage during catching and handling. The higher breast bleeding percentage in conventional broilers is probably caused by the large breast muscles lowering the center of gravity, which leads to reduced stability with an increased risk of accidental bruises (Wilhelmsson, 2016). In the present study, the percentage of DOAs for all broiler production systems was considerably lower than the 1% threshold of mortality during transport intervention used by the Netherlands Food and Consumer Product Safety Authority (NVWA).

**Table 4:** Carcass quality in the different broiler production systems.

	CONV	SGB1	SGB2	BLS	*P*-value
Hock burns (%)	5.23^a^	1.80^b^	1.28^b^	1.44^b^	<0.001
Footpad lesion score	35.0^a^	15.2^b^	15.3^b^	14.6^b^	0.002
Ammonia burns (%)	0.09	0.11	0.13	0.19	0.690
Scabby hips (%)	1.26	0.67	0.55	0.50	0.481
Wing hematomas (%)	2.47	1.71	1.92	2.05	0.412
Leg hematomas (%)	0.54^a^	0.28^b^	0.28^b^	0.27^b^	0.006
Breast bleedings (%)	0.38^a^	0.19^b^	0.19^b^	0.18^b^	<0.001
DOAs (%)	0.14^a^	0.09^b^	0.09^b^	0.10^b^	0.007
Total rejects (%)	2.05^a^	0.93^b^	0.55^b^	0.61^b^	<0.001

a-b Means within a column with no common superscript differences (*P* ≤ 0.05). ^1^CONV: conventional fast-growing broilers; SGB1: slow-growing broilers 1; SGB2: slow-growing broilers 2; BLS: better life 1 star broilers.

In the present study, no differences in the percentage of scabby hips and wing hematomas were found between conventional and slow-growing broilers. This is in contrast with a previous study that found more wing hematomas in slow-growing broilers than in fast-growing broilers (4.7% vs. 4.3%) (Gerritzen et al., 2019). They hypothesized that a higher percentage of wing hematomas in slow-growing broilers arise during catching and loading, which is probably due to the higher activity and stronger wing muscles of slow-growing broilers. The difference between the earlier and the present study could be caused by the difference in years of data collecting. In addition, it is hypothesized that, due to the increased knowledge about the behaviour and characteristics of slow-growing broilers under practical circumstances, the management, handling, and catching have probably been adjusted over time to prevent skin and bone damage.

No-thinning flocks showed a higher percentage of hock burns, wing hematomas, leg hematomas, breast bleedings, and total rejects and a higher footpad lesion score than the thinning flocks (Table 5). The higher percentage of hock burns and the higher footpad lesion score of no-thinning flocks are most likely due to the longer growth period (41.7 d vs. 34.5 d), which increases the risk of these abnormalities. The results regarding footpad lesions are in line with the study by de Jong et al. (2011). They also found that no-thinning flocks had more severe footpad lesions than thinning flocks. The difference in wing hematomas, leg hematomas, and breast bleedings between the thinning flocks and no-thinning flocks may have been caused by the difference in slaughter weight (1.97 vs. 2.64 kg; Table 2). Langkabel et al. (2015) found that broilers weighing approximately 2.5 kg had more wing hematomas than broilers weighing 1.9 kg (12 vs. 5%).

**Table 5:** Carcass quality of CONV thinning and no-thinning flocks.^1^

	CONV thinning flocks	CONV no-thinning flocks	*P*-value
Hock burns (%)	2.84^b^	5.23^a^	<0.001
Footpad lesion score	25.3^b^	35.0^a^	0.002
Ammonia burns (%)	0.14^a^	0.09^b^	0.690
Scabby hips (%)	1.60^a^	1.26^b^	0.481
Wing hematomas (%)	1.86^b^	2.47^a^	0.412
Leg hematomas (%)	0.45^b^	0.54^a^	0.006
Breast bleedings (%)	0.32^b^	0.38^a^	<0.001
DOAs (%)	0.143^a^	0.137^b^	0.007
Total rejects (%)	1.10^b^	2.05^a^	<0.001

a-b Means within a column with no common superscript differences (*P* ≤ 0.05).

1 CONV: conventional fast-growing broilers.

Thinning flocks showed a higher percentage of ammonia burns, scabby hips, and DOAs. Contrary to expectations, the percentage of ammonia burns was higher in thinning flocks than in no-thinning flocks. Because of the longer growth period, this was expected to be higher for no-thinning flocks. It is therefore hypothesized that the litter quality could improve after thinning, which could lead to recovery of the abnormality. It is hypothesized that the higher incidence of scabby hips among the thinning flocks occurred during handling and loading, likely due to individuals crawling over each other.

### Seasonal Effects on Carcass Quality

**Figure 2:**
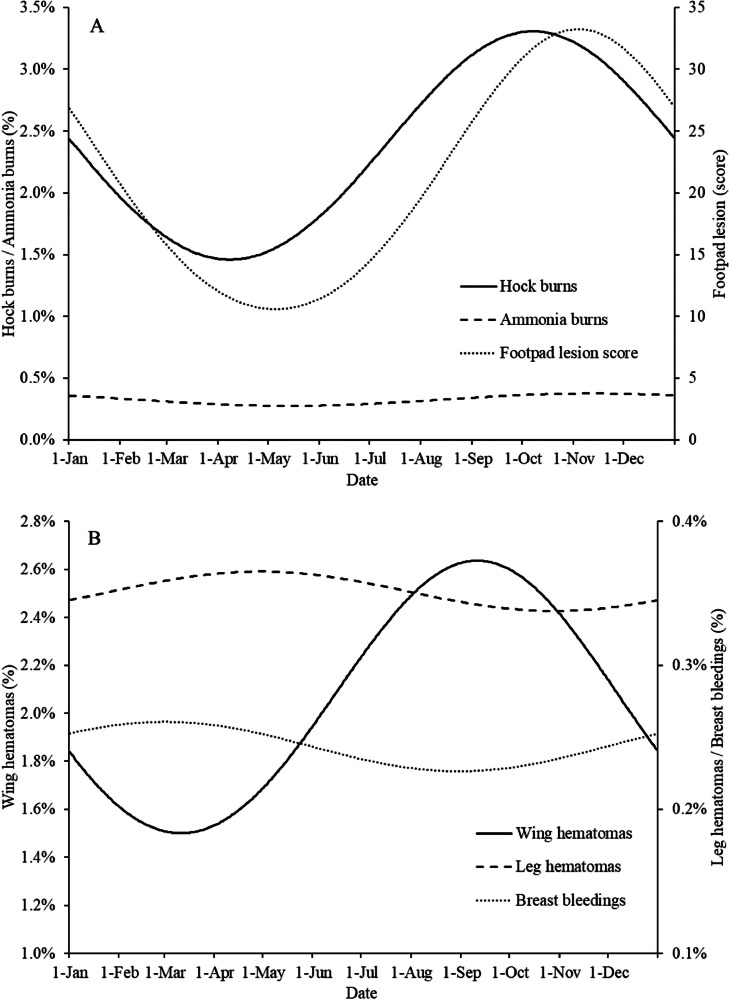

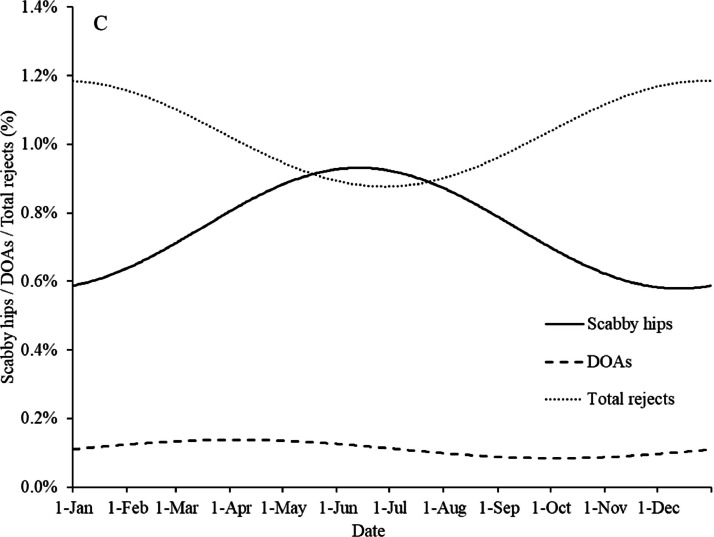
Estimated seasonal effects for hock burns (%), ammonia burns (%), footpad lesion score (A), wing hematomas (%), leg hematomas (%), breast bleedings (%) (B), scabby hips, DOAs, and total rejects (%) (C) of the broiler flocks. The X-axis represents the day of the year when DOCs were placed in the broiler house, starting on January 1 (d 1).

For all four production systems grouped significant effects (*P* < 0.05) of season were found on hock burns, footpad lesion score, ammonia burns, scabby hips, wing hematomas, DOAs, and total rejects (Table 6). A tendency (*P* = 0.097) to a seasonal effect was found for breast bleedings, and no seasonal effect was found for leg hematomas. The percentage of broilers with hock burns was the lowest when DOCs were placed in the broiler house in early April and the highest when they were placed in early October (Figure 2A). Footpad lesion score was the lowest when DOCs were placed in May–June and the highest when they were placed in November. This is consistent with studies from other regions, which also show that footpad lesions are more likely to occur during the wet and cold seasons (e.g., Haslam et al., 2007; Meluzzi et al., 2008). The percentage of broilers with ammonia burns shows a much less clear seasonal effect, with the lowest level in early June and the highest level in early November. Hock burns, ammonia burns, and footpad lesions are largely influenced by litter quality (Haslam et al., 2007), which in turn is strongly influenced by climatic conditions. Climate data from the years 2018 to 2020 showed that the outdoor relative humidity was higher and the outdoor temperature lower between September and March than between April and August (data not shown). These outdoor conditions result in a lower ventilation rate in the winter months with relatively high humidity, which can have negative effects on litter quality and thereby on hock burns, ammonia burns, and footpad lesion score (Ekstrand and Carpenter, 1998; Dawkins et al., 2004; Shepherd and Fairchild, 2010).

**Table 6:** Estimates of the fixed effects on broiler flock level in the statistical model of season per carcass quality parameter.

	*β_1_* Season (sinus)^3^	*Β_2_* Season (cosinus)^4^
Hock burns (%)	−8.53 * 10^−2^^1^	1.59 * 10^−2^
Footpad lesion score	−0.82^1^	0.67^1^
Ammonia burns (%)	−4.73 * 10^−3^^1^	4.23 * 10^−3^^1^
Scabby hips (%)	1.35 * 10^−2^	−2.97 * 10^−2^^1^
Wing hematomas (%)	1.09 * 10^−3^^1^	−1.06 * 10^−2^^1^
Leg hematomas (%)	1.71 * 10^−3^	8.84 * 10^−4^
Breast bleedings (%)	9.49 * 10^−4^^2^	2.15 * 10^−3^
DOAs (%)	4.31 * 10^−3^^1^	6.80 * 10^−4^
Total rejects (%)	−1.42 * 10^−3^	1.45 * 10^−2^^1^

1 Significant effect *(P* ≤ 0.05)

2 Tendency to an effect (*P* < 0.10); where no superscript is provided, no significant effect or trend was found.

3 *β1:* The seasonal sinus wave represents the spring–autumn variation (March 1 vs. September 1).

4 *β2:* The seasonal cosinus wave represents the summer–winter variation (January 1 vs. June 1).

There was also a seasonal effect on wing hematomas, and the incidence was the lowest when DOCs were placed in March and the highest when they were placed in September/October (Figure 2B). The explanation for this could be the increasing day length during catching. With increasing day length, the number of wing hematomas increases, because relatively more broilers were caught during daylight, resulting in more active birds that are more difficult to catch with a plausibly higher chance of damage and injuries. No seasonal effect was found for leg hematomas, and only a small tendency to a seasonal effect was found for breast bleedings.

A clear seasonal effect was found for scabby hips and total rejects, while there was a small seasonal effect on DOAs (Figure 2C). Scabby hips were found more in DOCs placed in broiler houses in June and July than in DOCs placed between November and January. This is because dawn (daylight) starts earlier in the summer months, which affects the light intensity and, consequently, the activity of the broilers during catching and loading (Nijdam et al., 2004), resulting in more birds huddling and piling up and therefore a higher risk of scabby hips. The total rejects show the opposite effect from scabby hips, with more broilers being rejected when DOCs were housed during the autumn and winter months than when they were housed during the spring and summer months. This is related to climatic conditions being worse in the autumn and winter months, with a higher risk of health problems and thus more rejects (Part et al., 2016). Part et al. (2016) also observed more ascites and abnormal colour/fever during the winter months than during the summer months. Although the differences were not large, the percentage of DOAs was slightly higher when DOCs were placed in the spring–summer period than when DOCs were placed in the autumn–winter period. This corresponds to a study by Knierim and Gocke (2003), who found 0.57% DOAs in the spring–summer period compared with 0.27% DOAs in the autumn–winter period. The higher percentage of DOAs during the summer period can be explained by the higher average temperature. The average percentage of DOAs (0.1%) in the present study is much lower than that in the study by Knierim and Gocke (2003). It is therefore hypothesized that the differences between the two studies are caused by the increased attention to and prevention of DOAs as a result of welfare legislation.

### Catching Method

Broilers which were mechanically caught had more ammonia burns, DOAs, and total rejects (Table 7). There was also a tendency toward a higher percentage of hock burns, leg hematomas, and breast bleedings in mechanically caught broilers. No differences were found in footpad lesion score, scabby hips, and wing hematomas between manual and mechanical catching. More ammonia burns and the tendency toward more hock burns in mechanical catching were unexpected, because these abnormalities already arise earlier during the growth period. These parameters have no relationship with the catching method but are directly affected by litter quality during the growth period (Berg, 2004). Therefore, a catching machine might have been used more frequently on farms with poorer litter quality, but this does not seem plausible given that the footpad lesion score did not differ between manual and mechanical catching. The tendency to a higher percentage of leg hematomas and breast bleedings in broilers caught with the catching machine may be related to the catching method. Previous experiments show inconsistent results regarding the effects of manual or mechanical catching on carcass quality in broilers. Knierim and Gocke (2003) found fewer hematomas, fractures, and dislocations of wings and legs in mechanically caught broilers than in manually caught broilers. In contrast, Musilová et al. (2013) found a higher incidence of catching damage in mechanically caught broilers (8.0%) than in manually caught broilers (6.8%). No effect of manual and mechanical catching on bruises and meat quality was observed by Nijdam et al. (2005). Gerritzen et al. (2019) found no differences in leg hematomas and breast bleeding but more wing hematomas and dislocations with mechanical catching than with manual catching. In the study by Gerritzen et al. (2019), however, only two flocks caught with a catching machine were compared with 28 flocks caught with manual catching. An earlier study by Delezie et al. (2006) found no differences in breast bleedings and leg hematomas between manual and mechanical catching; however, a lower percentage of wing hematomas was found with mechanical catching. The latter finding is in contrast to the findings of the present study, in which no differences were found in wing hematomas between manual and mechanical catching.

**Table 7:** Carcass quality by manual and mechanical catching.

	Manual	Mechanical	*P*-value
Hock burns (%)	2.32	3.73	0.072
Footpad lesion score	19.1	16.4	0.69
Ammonia burns (%)	0.33^b^	0.56^a^	0.029
Scabby hips (%)	0.77	0.79	0.82
Wing hematomas (%)	1.93	2.05	0.26
Leg hematomas (%)	0.35	0.40	0.087
Breast bleedings (%)	0.23	0.27	0.082
DOAs (%)	0.10^b^	0.15^a^	0.008
Total rejects (%)	1.02^b^	1.28^b^	0.042

a-b Means within a column with no common superscript differences (*P* ≤ 0.05).

A higher percentage of DOAs in mechanical catching was previously found by Knierim and Gocke (2003). They found 0.54% and 0.39% DOAs in mechanical and manual catching, respectively. Delezie et al. (2006) and Chauvin et al. (2011) also found a higher percentage of DOAs with mechanical catching and loading than with manual catching and loading. Delezie et al. (2006) suggested that this may have been caused by birds that were unfit or already dead before depletion and that had not been removed by the farmer before loading, whereas these broilers would have been removed during manual catching by the catching team.

### Relative Size of the Variance Components for the Different Carcass Quality Parameters

Table 8 presents the estimations of the relative size of the variance components for the different carcass quality parameters. A relatively high estimate of a variance component for a certain quality parameter indicates what the most important influencing factors are. That means that improvements in these factors can have a relatively high positive effect on that specific parameter. The table shows that after correction for the influences of CONV thinning flocks, system, season, slaughter location, catching method, and the interaction between slaughter location and season, there are virtually no constant differences (influences) between broiler farms. This means that this analysis does not show that a specific broiler farm has consistently performed better or worse than other broiler farms for a certain quality parameter. An exception is the characteristic footpad lesions, for which the variance component is almost 18% of the total variance. Broiler farms place DOCs on different dates throughout the whole year in several houses. For most characteristics (except DOAs: 1.7%), differences between starting dates were found. This may be due to variation in the quality of DOCs, but possibly (coincidentally) different circumstances at the broiler farm (or during transport) can also play a role. The house-specific starting date concerns differences between broiler houses with the same starting date. It is remarkable that for many traits, there do not seem to be any significant differences in house-specific starting dates. The house-specific starting date does have a relatively large influence on total rejects. More than 18% of the total variance of the total rejection parameter can be attributed to the house-specific starting date. In addition, the house-specific starting date influences the occurrence and/or prevalence of footpad lesions; almost 11% of the total variance can be attributed to this factor. This is not surprising, since the quality of DOCs and the occurrence of diseases and/or digestion problems can have a major impact on the carcass quality at slaughter age. The influence of the feed company on the various quality characteristics is very limited. In other words, there are virtually no constant differences between feed companies for most quality traits, with the exception of hock burns and footpad lesions, although the variance components are relatively small (more than 5% and almost 3% of the total variance, respectively).

**Table 8:** Estimations of the relative size of the variance components for the different carcass quality parameters.^1^

	Hock burns (%)	Footpad lesion score	Ammonia burns (%)	Scabby hips (%)	Wing hematomas (%)	Leg hematomas (%)	Breast bleedings (%)	DOAs (%)	Total rejects (%)
*ε_ik_* System/slaughter location interaction	0.3	0.2	0.0	23.4	2.8	4.5	0.0	0.0	0.0
*ε_l_* Farm	1.2	17.7	0.0	0.0	0.0	0.0	0.0	0.0	1.2
*ε_kl_* Farm/slaughter location interaction	2.2	1.1	2.7	2.0	1.6	0.4	0.2	1.0	0.5
*ε_lp_* House within farm	0.7	3.5	0.5	0.0	0.0	0.0	0.1	0.0	0.7
*ε_β1l_* Starting season/farm interaction	2.1	0.7	0.0	0.0	0.0	0.2	0.0	0.0	1.1
*ε_lq_* Starting date (within farm)	24.4	27.5	16.5	15.2	5.5	6.9	5.0	1.7	29.7
*ε_lpq_* House-specific starting date	0.0	10.5	0.0	0.0	0.0	0.0	0.0	0.0	18.4
*ε_io_* Breed within system	0.5	1.4	0.1	0.8	5.7	0.8	0.4	0.1	1.7
*ε_im_* Feed company within system	5.2	2.7	0.2	0.6	0.1	0.1	0.0	0.2	0.8
*ε_in_* Hatchery within system	0.7	1.6	20.8	0.2	0.0	0.0	0.2	0.2	4.6
*ε_v_* Catching team	0.2	1.5	0.1	0.5	0.1	0.0	0.0	0.2	0.1
*ε_β2v_* Starting season/catching team interaction	0.4	2.0	0.2	0.0	0.0	0.1	0.0	0.1	0.5
*ε_kw_* Slaughter location/week interaction	2.8	8.7	0.0	9.2	20.4	10.8	0.0	0.0	2.3
*ε_il_* Farm differences within system	11.0	6.4	7.2	1.0	0.7	0.0	0.0	7.9	5.4
Residual variation (delivery within *ε_lpq_)*	48.4	14.6	51.7	47.2	63.2	76.3	94.1	88.6	32.9

1 The rows “starting date within farm” (*ε*_lq_), “house-specific starting date” (*ε*_lpq_), and the residual term (“delivery within *ε*_lpq_”) indicate the relative variance in broiler flock carcass quality that cannot be explained by average seasonal effects (see Table 5); production system (see Table 4), mechanical catching (see Table 6), and slaughter location effects; average effects of farm (*ε*_l_), house within farm (*ε*_lp_), and catching team (*ε*_v_); average effects of breed (*ε_io_)*, feed company(*ε_im_)*, and hatchery (*ε_in_)* (all within a production system); average effect of the specific combinations of farm, slaughter location, and production system; average effect of specific seasonal tendenc of farm (*ε_β1l_)* and catching team (*ε_β2v_)*; and average effect of the specific combination of slaughter location and week in the total period (*ε*_kw_). The row “starting date within farm” (*ε*_lq_) includes the incidental effects linked to specific start-up conditions on the broiler farm and the specific quality of the DOC batch. Occasional differences between houses filled on the same farm on the same day (*ε*_lpq_) include the incidental effects of house-specific conditions.

## CONCLUSIONS

Production systems with slow-growing broilers had a lower slaughter weight, ADG, first-week mortality, total mortality, and footpad lesion score and a lower incidence of hock burns, leg hematomas, breast leg hematomas, DOAs, and total rejects than the CONV production system. There was no difference in carcass quality and welfare indicators between the three production systems with slow-growing broilers. SGB1 broilers had a higher ADG than SGB2 and BLS broilers. For all four production systems grouped, several seasonal effects were found on production performance and carcass quality. Slaughter weight and ADG were the lowest when DOCs were placed in the house in March/April and the highest when they were placed in September/October. Clear seasonal effects with more hock burns, a higher footpad lesion score, and more wing hematomas were found in the autumn months compared with the spring months. More scabby hips and fewer total rejects were found during the summer months than during the winter months. Thinning flocks had more scabby hips, ammonia burns, and DOAs than no-thinning flocks, whereas no-thinning flocks had more hock burns, footpad lesions, wing hematomas, leg hematomas, breast hematomas, and total rejects. Mechanically caught broilers had more ammonia burns, DOAs, and total rejects, and tended to have more leg hematomas and breast bleedings than manually caught broilers. The relative size of the variance components, except the starting date, for the different carcass quality parameters was relatively low.

In conclusion, this study showed that production systems with slow-growing broilers had a lower first-week mortality, total mortality, DOAs and better scores of most carcass quality parameters than conventional broilers, while season had an important impact on most parameters for all four production systems. This indicates that broilers in slow-growing production systems have better scores on the health domain of animal welfare.
